# Grand Challenges: Integrating Maternal Mental Health into Maternal and Child Health Programmes

**DOI:** 10.1371/journal.pmed.1001442

**Published:** 2013-05-07

**Authors:** Atif Rahman, Pamela J. Surkan, Claudina E. Cayetano, Patrick Rwagatare, Kim E. Dickson

**Affiliations:** 1 University of Liverpool, Institute of Psychology, Health & Society, Child Mental Health Unit, Alder Hey Children's NHS Foundation Trust, Mulberry House, Eaton Road, Liverpool, United Kingdom; 2 Social and Behavioral Interventions Programme, Department of International Health, Johns Hopkins Bloomberg School of Public Health, Baltimore, Maryland, United States of America; 3 Mental Health Programme, Ministry of Health, Belmopan, Belize; 4 Department of Mental Health, University Teaching Hospital of Kigali, Nyarugenge District, Rwanda; 5 Health Section, UNICEF, United Nations Plaza, New York, United States of America

## Abstract

In the second article of a five-part series providing a global perspective on integrating mental health, Atif Rahman and colleagues argue that integrating maternal mental health care will help advance maternal and child health.

*Please see later in the article for the Editors' Summary*

**Summary Points**

- Integrating maternal mental health care will help advance maternal and child health (MCH) status.
- Misconceptions regarding maternal depression are obstacles to the integration of mental health initiatives in MCH programmes.
- Myths about maternal mental health include the beliefs that: maternal depression is rare, not relevant to MCH programmes, can only be treated by specialists, and its incorporation into MCH programmes is difficult.
- Collaboration between policy makers in mental health and those in MCH is imperative for action that will advance maternal and child health status.
- Strategies to improve maternal mental health have to be linked to broader development goals, including poverty reduction and gender empowerment.
- This paper is the second in a series of five articles providing a global perspective on integrating mental health.

*This is one article in a five-part series providing a global perspective on integrating mental health.*

## Introduction

Over the last decade, a number of influential organisations have called for the integration of mental, neurological and substance use (MNS) disorders into large scale public health programmes [1]. Although progress at the implementation level has been slow, the development of a number of evidence-based, potentially scalable interventions in the MNS field [2] provides new impetus to develop strategies for integration with broader programmes.

The World Health Organization defines maternal mental health as “a state of well-being in which a mother realizes her own abilities, can cope with the normal stresses of life, can work productively and fruitfully and is able to make a contribution to her community” [3]. Globally, in women of child-bearing age, depression accounts for the largest proportion of the burden associated with mental or neurological disorders [4],[5] (Box 1). In addition to the economic and human costs of maternal depression, children of depressed mothers are at risk for health, developmental, and behavioral problems [6], thereby contributing to inter-generational disadvantage that accumulates throughout the life span. Addressing mental health concerns such as maternal depression could play an important role in achieving the Millennium Development Goals set by the United Nations (three out of the eight goals refer specifically to women and children) [7]. However, mental health care remains conspicuous by its absence in large scale global maternal and child health (MCH) programmes. Critical obstacles include a number of misconceptions, or *myths*, about maternal depression. These myths have important consequences, depriving many mothers of their basic right to health and well-being, and preventing their children from reaching their full developmental potential.

**Box 1. Public Health Dimensions of Maternal Depression**

- The second leading cause of disease burden in women worldwide, following infections and parasitic diseases
- Presents with low mood, loss of interest or pleasure, feelings of guilt or low self-worth, disturbed sleep or appetite, low energy, and poor concentration – symptoms that profoundly affect maternal functioning and role
- Can become chronic or recurrent and leads to substantial impairments in the mother's ability for child care
- Has long-term negative effects on infant physical and cognitive development
- Can lead to suicide – a leading cause of mortality in women of child-bearing age

In this paper, one in a five-part series, we examine the state of the field of maternal mental health and consider the challenges as well as the opportunities for integration into MCH programmes.

## Misconceptions about Maternal Mental Health

### Myth 1: Maternal Depression is Rare

A common perception is that depression is a construct of affluent Western societies that is infrequent or non-existent in traditional communities. Perinatal depression (during pregnancy and in the year after birth) has been reported in all cultures. Rates vary considerably, but the average in high-income countries about 10% to 15% [8]. A recent meta-analysis shows that the rates in low- and middle-income countries (LMICs) are even higher, ranging from 18% to 25% [9]. Contributing factors related to depression in women include poverty and persistent poor health; a poor relationship with a partner (including intimate partner violence); insufficient practical or emotional support from the family; few confiding relationships and lack of assistance in crises; social adversity; limited control or participation in financial decisions or reproductive health, including crowded living conditions and lack of employment; and coincidental adverse life events, such as financial difficulties, unwanted pregnancy, or illness in the child [6],[9].

### Myth 2: Maternal Depression is Not Relevant to MCH Programmes

Depression is the leading contributor to the global burden of disease (more years of life lived with disability, reduced productivity including unemployment, increased physical illness, increased health expenditure, impact on families and caregivers, and premature mortality) [5]. Strong evidence shows that maternal depression, especially amongst those experiencing social disadvantage, is linked to poor outcomes in infants. Although some studies have not found associations or only found them in subgroups [10]–[13], others show strong and independent associations with pre-term birth [14], low birthweight [14] and undernutrition in the first year of life [15], higher rates of diarrheal diseases, and early cessation of breastfeeding [6]. Depressive symptoms in low-income pregnant women and mothers have been associated with use of tobacco, alcohol, and illicit drugs [16]; adverse birth outcomes [2]; chronic health problems [17]; low maternal self-esteem [18],[19]; and parenting difficulties [20]. The strength of associations vary across studies, however, which could be attributable to social and cultural differences between populations as well as differences and quality of the study designs. Notably, one review indicated that children of depressed mothers have significantly poorer long-term cognitive development; have higher rates of antisocial behaviour, hyperactivity and attention difficulties; and more frequently experience emotional problems [6]. Nonetheless, despite this adversity, many children remain physically healthy and develop normally, demonstrating resilience of maternal care and child development.

Suicide, strongly associated with depression, is a leading contributor to maternal mortality globally. Maternal depression and suicide are strongly associated with gender-based violence; i.e., more than one fifth of women who have experienced violence attempt suicide [21]. Suicidality – thoughts of suicide or actual self-harm – occurs in up to 20% of mothers in LMICs and from 5% to 14%in high-income countries [22], and rates are even higher in displaced populations [23]. It is common in young women and associated with abuse and stress that might be caused by such events as unwanted pregnancy.

### Myth 3: Only Specialists Can Treat Maternal Depression

“Talking” or psychological therapies typically are a front-line treatment for depression. Only a minority of individuals with depression will require anti-depressant medication. Psychological therapies include cognitive behavioral therapy, interpersonal therapy, supportive therapy, and group therapy. Systematic reviews from high-income countries provide evidence of the effectiveness of both psychological therapies and pharmacotherapy in the treatment of perinatal depression [24].

Over the last decade, the evidence for the effectiveness of non-mental health specialist-led interventions (e.g., involving nurses, health visitors, and midwives) in high-income countries has been building [25]–[30]. Efforts to improve maternal mental health through such interventions in developing countries are promising. Several successful randomized controlled trials have been delivered by lay health workers, a critical resource in settings in which formally trained health professionals are often scarce. Studies where maternal mental health was delivered effectively by non-specialists are summarized in Table 1 [31]–[33].

**Table 1:** Effective Interventions for Maternal Mental Health Integrated into Maternal and Child Health.

Level of Integration	Author/Location	Intervention	Design and Participants	Outcome Measures	Main Findings
Integrated into a nutrition and positive parenting programme	Baker-Henningham et al. 2005 [31] Location: In nutrition clinics in Kingston, St Andrew, and St Catherine, Jamaica.	• Half-hour weekly home visiting intervention to improve mothers' knowledge and child-rearing practices and parenting self-esteem; encourage age-appropriate play activities and responsive feeding; empathic listening and praise.• Control group received standard health and nutrition care at the clinics.	Randomised controlled trial.139 mother–infant dyads - child aged 9 to 30 months; weight for age −1.5 *z*-scores of national reference and −2 *z*-scores in prior 3 months.	Measured at 1 year post intervention.Maternal mood: Culturally modified version of the CES-D to assess maternal depression.Child development: Griffiths Mental Development Scale subscales assessing: locomotor, hearing and speech, hand–eye coordination, and performance development to give a global developmental quotient (DQ).	Maternal mood: Decline in depressive symptoms in intervention, but not control group mothers (*b* = −0.98; 95% CI −1.53 to −0.41); mothers receiving 40–50 home visits had greatest decline in depressive symptoms (*b* = −1.84; 95% CI −2.97 to −0.72).Child development: Final maternal depression and final DQ correlated in boys (*p*<0.05), but not in girls.
Integrated into a community health programme	Rahman et al. 2008 [32] Location: Union Council clusters in two sub-districts: Gujar Khan and Kallar Syedan in a rural area 65 km southeast of Rawalpindi city in Pakistan.	• 16 home visits: 4 antenatally and 12 until 1 year postnatally using the Thinking Health Programme (THP), a manualised intervention incorporating cognitive and behavioural techniques.• Control group received enhanced routine care: the same number and schedule of home visits, but from an untrained community health worker.	• Cluster randomised controlled trial.• Women aged 16–45 years, in the third trimester of pregnancy, meeting SCID criteria for DSM-IV major depressive episode (*n* = 903).	Measured at 6 months and 1 year post intervention.Maternal moodHDRS and SCID at 6 and 12 months postpartum to assess maternal depression.Infant health and development: Infant anthropometry: weight and length; number of diarrhoeal episodes in previous fortnight and immunisation status of the infant*;* time dedicated to infant play.	Maternal mood: After adjusting for covariates, mothers in the intervention group (a) Were less likely to be depressed at 6 months postpartum (23% vs 53%, AOR 0.22, 95% CI 0.14 to 0.36, *p*<0.0001); (b) were less likely to be depressed at 12 months postpartum (27% vs 59%, AOR 0.23, 95% CI 0.15 to 0.36, *p*<0.0001); and (c) had better perceived social support at 6 (AMD 6.71, 95% CI 3.93 to 9.48, *p*<0.0001) and 12 months (AMD 7.85, 95% CI 5.43 to 10.27, *p*<0.0001).Infant health and development: (a) Infants of intervention group mothers had fewer episodes of diarrhoea at 12 months (AOR 0.6, 95% CI 0.39 to 0.98, *p* = 0.04); and were more likely to be fully immunised (AOR 2.5, 95%CI 1.47 to 4.72, *p* = 0.001); (b) Both parents dedicated time to playing with the baby (AOR mothers 2.4, 95%CI 2.07 to 4.01, *p*<0.0001; AOR fathers 1.9, 95%CI 1.59 to 4.15, *p* = 0.0001).
Integrated into a child development programme	Cooper et al. 2009 [33] Location: Khayelitsha, a periurban settlement outside Cape Town, South Africa.	16 home visits – 2 antenatally and 14 until 5 months postnatally –using an adaptation of the Health Visitor Intervention Programme incorporating principles of WHO's Improving the Psychosocial Development of Children programme to: (a) Enhance maternal sensitivity and responsiveness to and interactions with her baby; and (b) compare with group that received only standard health care.	All women, in third pregnancy trimester, identified systematically in regular home visits (*n* = 449).	Mother-infant interaction: At infant age 6 months, video tapes of 10 minutes of free play independently scored to assess maternal sensitivity and intrusiveness.Infant attachment: At infant age 18 months, the Strange Situation Procedure.Maternal depression: 6 months postpartum SCID interviews, which incorporated the EPDS.	Maternal depression: Lower prevalence of depression in intervention than in control group at 6 and 12 months, but differences not significant; EPDS scores lower in intervention than control groups at both assessment points, but difference only significant (*p* = 0.04) at 6 months.Mother–infant interaction: Intervention group significantly more sensitive and less intrusive in interactions with their infants at both 6 and 12 months, all *p*<0.05.Infant attachment: More infants in the intervention than in the control group were securely attached (OR 1.70, *p*<0.029).

Rahman A, Fisher J, Waheed W. (2009). Maternal mental health interventions in low and middle income countries: A systematic review of the evidence. Unpublished manuscript. Department of Mental Health and Substance Abuse, World Health Organization, Geneva.

AMD, adjusted mean difference; AOR, adjusted odds ratio; CES-D, Center for Epidemiologic Studies Depression Scale; DQ, developmental quotient; EPDS, Edinburgh Postnatal Depression Scale; HDRS, Hamilton Depression.

Rating Scale; OR, odds ratio; PPVT-R, Peabody Picture Vocabulary Test – Revised; SCID, Structured Clinical Interviews for DSM IV Diagnoses; NBAS, Neonatal Behavioural Assessment Scale.

### Myth 4: It is Not Possible to Integrate Mental Health Care into MCH Programmes

Policy makers fear that mental health interventions will divert the energies of health care staff and dilute the impact of other “priority” interventions. This view fails to take into account the holistic nature of health and erroneously propagates the defunct theory of “mind-body” dualism. Most current evidence demonstrates the inter-connectedness of physical and mental health and suggests that integrated interventions can achieve synergistic results [4].

Examples of community-based trials with a maternal mental health component integrated into a MCH programme [31]–[33] (Table 1) and a case study demonstrating that the screening and management of maternal mental disorders can be integrated successfully into an existing health system at a facility level [34] (Box 2) build a strong case for integration of mental health care into MCH programmes. The studies in Table 1, all examples of community-based interventions, have common elements that make them more amenable to integration. Some of these elements are described in the formative research to develop the Thinking Healthy Programme in Pakistan [35]: (a) The intervention has a child-focus, thus ensuring buy-in from the families, and avoiding stigmatization; (b) It is woven into the routine work of the community health worker so it is not seen as an extra burden but, being delivered alongside normal duties, is seen to support his or her routine work; and (c) The intervention itself is simple and culturally appropriate and employs robust and supportive training and supervision processes. The Thinking Healthy Programme has been further adapted so that it can be used *universally* for all women rather than only depressed women.

**Box 2. Case Study of Integration of a Stepped Care Intervention with a Facility-based MCH Programme in Urban South Africa [33]**

Neither routine screening nor treatment of maternal mental disorders are available in primary care settings in South Africa. The Perinatal Mental Health Project, based at the Mowbray Maternity Hospital in the Western Cape Province of South Africa, developed a stepped care intervention for maternal mental health that is integrated into antenatal care. Mowbray Maternity Hospital is a secondary level maternity hospital, linked to the University of Cape Town, and is located centrally within the city. The Perinatal Mental Health Project services are based at the hospital within the Midwife Obstetric Unit, which provides a primary level antenatal clinic. Midwives at the Midwife Obstetric Unit are trained to screen women routinely for maternal mood disorders during their antenatal visits. Women in whom screening yields positive findings are referred to on-site counsellors who also act as case managers. When specialist intervention is indicated, women are referred to an on-site psychiatrist. The Perinatal Mental Health Project works directly with facility managers and health workers through collaborative partnerships, focusing on problem solving and capacity development in the primary health care system. Over a 3-year period, 90% of all women attending antenatal care in the maternity clinic were offered mental health screening with 95% uptake. Of those screened, 32% qualified, of which 47% received counselling through the programme.

A number of important lessons have been learned from this programme: (a) maternity health workers may be trained to screen for and refer women with mental distress in low-resource primary care settings; (b) training programmes that address and support the mental health needs of health workers may help staff to manage their workload and prevent compassion fatigue and “burn out”; (c) on-site screening and counselling fosters the establishment of efficient referral mechanisms and access to mental health care often lacking in maternity settings in LMICS; (d) on-site, integrated mental health services increase access for women who have scarce resources and competing health, family, and economic priorities; e) coordinating mental health visits with subsequent antenatal visits further facilitates access for women with insufficient resources.

The challenges to integrating maternal mental health interventions at scale remain substantial. In LMICs, the main barriers include resource allocation, lack of human resource, and weak health systems [36],[37]. Globally, a major barrier remains the social exclusion and negative attitudes attached to mental illnesses – a problem as much of the public health professionals as of the communities they serve.

## The Way Forward

In 2011, the Grand Challenges in Global Mental Health, an initiative of a consortium of researchers, advocates, and clinicians, announced priorities for improving the lives of people with mental illness around the world and called for urgent action and investment [38]. The principles underlining the setting of such priorities are to use a life-course approach, system-wide approaches to address suffering, and evidence-based interventions, and to understand the environmental context in which illness occurs and services are delivered. We believe these principles are not limited to mental health but are of direct relevance to all of MCH. Long-term follow-up studies from high-income countries show that the offspring of depressed mothers are at a higher risk of poor mental health and social impairment from early childhood through to adolescence and adulthood, and have an increased risk of mortality from medical causes [38]. While such studies have not been conducted in LMICs, the impact of maternal depression through the life course is likely be even greater, given the strong associations with infant low birthweight and undernutrition. This paper highlights evidence-based interventions that can serve as models for integration into existing health systems to improve not only maternal depression but also immediate perinatal and long-term infant health and developmental outcomes, with likely benefits throughout the life course. The immediate needs are to make policy makers, planners, and politicians aware of the missed opportunity of integrating these interventions into mainstream MCH programmes and to direct both research and implementation funds to meet the challenges of scaling up these promising approaches.

The exercise of defining Grand Challenges provides a framework for future steps in this direction [39]. The Challenges especially relevant to maternal depression in the global MCH context are:

- Enhance collaboration between MCH and mental health programmes, researchers, and practitioners
- Develop ways to integrate screening and core packages of mental health services into routine primary health care (e.g., antenatal visits) and establish effective referral mechanisms
- Further develop effective treatments for use by non-specialists, including lay health workers with minimal training
- Address stigma related to mental illness that could impede the integration of mental health into MCH programmes
- Increase capacity in low- and middle-income countries by creating regional centers for mental health research, education, training, and practice that incorporate the views and needs of local people
- Develop sustainable models to train and increase the number of culturally and ethnically diverse lay and specialist providers to deliver evidence-based services
- Strengthen the mental health component in the training of all health care personnel
- Redesign health systems to integrate maternal depression with other chronic disease care, and create parity between mental and physical illness in terms of investment into research, training, treatment, and prevention
- Incorporate a mental health component into international MCH aid and development programmes

The responsibility to meet these challenges is not the sole duty of the mental health community, but rather it should be shared equally by the MCH researchers and funders. But where does one begin? Firstly, gain recognition at the highest international and national policy forums that mental health and well-being is a generic component of MCH that does not compete with MCH programmes but instead complements them. Secondly, enhance the training and supervision of MCH community and primary care personnel so that they can recognise and treat psychosocial distress and depression in women, enabling them to be more effective health workers. Thirdly, adapt effective interventions to local contexts and strengthen systems of supervision, referral, and continued training at the primary, secondary, and tertiary levels so that the community component is well supported. Finally, it is imperative to invest in research and implementation programmes so that these approaches are refined and scaled-up, leading to improved outcomes of all MCH programmes.

Along with efforts to integrate maternal mental health care into MCH programmes comes the need to pay attention to factors contributing to the high rates of maternal depression, especially in resource-poor settings. Epidemiologic data indicate that the origins of depression in women can be traced to the social circumstances of their lives [6],[9]. As Desjarlais and colleagues [40] eloquently pointed out: “hopelessness, exhaustion, anger and fear grow out of hunger, overwork, violence and economic dependence. Understanding the sources of ill health for women means understanding how cultural and economic forces interact to undermine their social status. If the goal of improving women's well-being from childhood through old age is to be achieved, *healthy* policies aimed at improving the social status of women are needed along with health policies targeting the entire spectrum of women's health needs”. Integration of maternal mental health into the MCH agenda can provide a universally acceptable “window of opportunity” for creating *healthy* policies, from education to economic empowerment to legal and political mechanisms that enhance the status of women.

The evidence presented in this paper shows that many outcomes that contribute to infant mortality, such as undernutrition, diarrhoeal disease, immunization, and breastfeeding uptake, have direct associations with maternal mental health. By narrowly fixating on mortality and morbidity targets, and relegating psychosocial well-being to be a peripheral goal, the MCH community is missing an important opportunity and, in the process, depriving millions of mothers and children of their basic right to health. This situation needs to be remedied immediately and urgently.

## Abbreviations

LMICs: low- and middle-income countries
MCH: maternal and child health
